# Using Cross-Sectional Data to Identify and Quantify the Relative Importance of Factors Associated with and Leading to Food Insecurity

**DOI:** 10.3390/ijerph15122620

**Published:** 2018-11-22

**Authors:** Alison Daly, Christina M. Pollard, Deborah A. Kerr, Colin W. Binns, Martin Caraher, Michael Phillips

**Affiliations:** 1 Faculty of Health Science, School of Public Health, Curtin University, GPO Box U1987, Perth 6845, Western Australia, Australia; C.Pollard@curtin.edu.au (C.M.P.); D.Kerr@curtin.edu.au (D.A.K.); C.Binns@curtin.edu.au (C.W.B.); 2 Centre for Food Policy, City University of London, Northampton Square, London EC1V 0HB, UK; m.caraher@city.ac.uk; 3 Harry Perkins Institute for Medical Research, University of Western Australia, Perth 6009, Western Australia, Australia; michael.phillips@perkins.uwa.edu.au

**Keywords:** food insecurity, monitoring, surveillance, determinants, path diagram

## Abstract

Australian governments routinely monitor population household food insecurity (FI) using a single measure—‘running out of food at least once in the previous year’. To better inform public health planning, a synthesis of the determinants and how they influence and modify each other in relation to FI was conducted. The analysis used data from the Health & Wellbeing Surveillance System cross-sectional dataset. Weighted means and multivariable weighted logistic regression described and modelled factors involved in FI. The analysis showed the direction and strength of the factors and a path diagram was constructed to illustrate these. The results showed that perceived income, independent of actual income was a strong mediator on the path to FI as were obesity, smoking and other indicators of health status. Eating out three or more times a week and eating no vegetables more strongly followed FI than preceded it. The analysis identified a range of factors and demonstrated the complex and interactive nature of them. Further analysis using propensity score weighted methods to control for covariates identified hypothetical causal links for investigation. These results can be used as a proof of concept to assist public health planning.

## 1. Introduction

Food security exists “when all people, at all times, have physical, social and economic access to sufficient safe and nutritious food that meets their dietary needs and food preferences for an active and healthy life” [1]. Conversely, food insecurity (FI) is the “limited or uncertain availability of nutritionally adequate and safe foods or the limited or uncertain ability to acquire acceptable food in socially acceptable ways” [2], and is increasing in developed countries [3]. FI is adversely related to diet quality [4,5,6,7,8,9,10] and has been associated with the double burden of malnutrition, including undernourishment and obesity [11,12,13] and additionally it has been associated with poor mental health and socioeconomic disadvantages [14,15,16,17,18,19,20]. 

The complexity and impact of FI has been acknowledged and there is an growing amount of attention being directed to its determinants and how they influence and modify each other, calling for a systemic food system response [21]. FI is a problem of social and economic disadvantage, of which ‘running out of food’ due to insufficient money is only one component [22]. The complex nature of decisions about food is constrained by both physical access and choice [23], underpinned by the Food and Agricultural Organization’s four pillars of availability: Access, utilization, stability and sustainability [24,25,26]. There is growing consensus regarding the need to focus on and better integrate social and structural factors when developing policies and interventions to improve public health in high income countries [27,28]. Evidence that is accessible to policy makers in the increasingly interrelated and complicated health policy area requires new approaches to research types and analyses [29].

The prevalence of FI in Australia, based on a 2001 review of the literature, showed that the rates were higher among the following groups: Families living with low or unstable incomes, those in remote areas, Aboriginal and Torres Strait Islander people, the unemployed, those living in rental households, single parents, those who were never married, separated or divorced, young adults and the elderly, asylum seekers and migrants, and people with disabilities [30]. FI directly impacts short and long-term health status, contributing to poor physical and psychological health outcomes and Australian health care costs [30]. The paradoxical relationship between FI and obesity has also been demonstrated, also significantly contributing to increasing health care costs [31,32,33,34].

Governments are increasingly encouraged to monitor FI, its determinants, mitigating actions, and their effectiveness [35]. Some countries, including Australia, do measure and report the prevalence of FI, including its severity and/or its determinants [36,37,38,39], but not routinely. While FI measures are continuously being evaluated and validated to come up with more accurate estimates of FI, the evaluation of the measures generally only contain limited references to determinants [5,40,41,42]. The severity of FI’s effects, as well as its determinants and associated factors are important information used to inform public health planning. Currently there is little recognition among health or social services policy makers regarding the extent of the problem among some population sub-groups, nor the impact of sociodemographic determinants. 

This study uses a cross-sectional self-reported dataset (the Western Australian Department of Health’s *Health & Wellbeing Surveillance System* 2009–2013, *(HWSS))* to construct a path diagram of variables leading to ‘running out of food’ at least once in the previous year because of insufficient money. The analysis evaluates the relative importance of variables associated with FI. Specifically, the study aims to: Conduct an analysis to evaluate the relative importance of a range of associated variables with FI; use the results of the analysis to construct a path diagram to FI; propose hypothetical causal paths to and from FI; and suggest how future research and policy can be developed more effectively. 

## 2. Materials and Methods 

### 2.1. Sample and Measures 

The HWSS cross-sectional computer-assisted telephone interview survey has measured health and wellbeing indicators (including risk factors) since 2002. Stratified samples by area were drawn from the statewide telephone book *Electronic White Pages* with geocoded addresses. The average participation rate was 90.2%. The 2009 to 2013 dataset, with a total of 21,710 adults aged 18–64 years was analysed. Data were pooled and weighted for probability of selection using iterative proportional fitting with marginal totals for the distribution of Western Australia (WA) residents in 2011 by age, sex and geographic area. The Department of Health in Western Australia datasets are not publicly available. The HWSS was granted ethics approval from the Western Australia Department of Human Research Ethics Committee (HREC 2011/65). 

The sociodemographic variables used in this study were: Age, gender, highest level of education attained, living arrangements, area of residence, annual household income (AUD$), perceived discretional income, country of birth employment status and the geographic area based index that reflects socioeconomic advantage and disadvantage (SEIFA) [43]. Self-reported body weight and height, using a correction for over-reported height and under-reported weight [44], was used to estimate the Body Mass Index (BMI) of each respondent. Health-related variables included the self-assessment of: General health, comparison of health with a year prior, psychological distress (using the Kessler 10 index) [45], health risk factors and whether or not the respondent had these variables diagnosed by a doctor. The indicators of self-reported dietary behaviour included daily fruit, vegetable, and low-fat milk intake, as well as weekly take-away food consumption. 

### 2.2. Analysis 

The strategy adopted for this analysis was to develop a path diagram to describe a hypothetical model for the network of associations that describe running out of food and its consequences. This method has been used previously [46]. Usually this approach would use a structural equation model (SEM) but the outcome (running out of food or not) was dichotomous, meaning that SEM could not be used. Logistic regression analyses were conducted with a reference group of respondents who did not run out of food. The variables listed in Table 1 were statistically significant at *p* ≤ 0.1 and were entered into weighted multivariable logistic regression analyses. While some variables were collected as continuous (e.g., age, K10, fruit, vegetables and physical activity estimates) values, we grouped them based upon accepted guidelines for Australian adults where possible. This was done to avoid assumptions of linearity. Both two way and three way interaction terms between the variables were tested on the final multivariable regression models. Bootstrapping (100 repetitions) produced final model estimates with robust measures used to estimate standard errors for the regression analyses. Results at *p* < 0.05 were considered to be statistically significant and kept in the model. Goodness of fit was assessed using the Hosmer–Lemeshow test. Diagnostic post-estimation tests, including tests for multicollinearity were conducted. The regression results were used to conduct a path diagram where the Bayesian Information Criteria (BIC) [47] was used to determine whether or not an association preceded or came after ‘running out of food’. The ordering with the lowest BIC was used to determine the direction of the association. A difference in BIC of 10 or more (considered a very strong indicator) was the minimum value when deciding upon direction of effect. This corresponded to a *p* value of <0.0004 [48]. The multivariable model was modified to incorporate this information and the path diagram was constructed to reflect the results of the final model. Propensity scores were used to control for potential confounding from the covariates in iterative propensity score weighted logistic regression analyses for four variables. The four variables tested were income, discretional income, eating fast food three or more times a week and eating no vegetables. These four independent variables were operationally defined as variables in the path leading to the outcome of either ‘running out of food’ or not [49]. Each of these variables were tested for hypothetical causality. All analysis was conducted using Stata 13.1 [50]. 

## 3. Results

A total of 709 respondents reported ‘running out of food’ at least once in the previous twelve months and couldn’t afford more (unweighted prevalence = 3.3%; weighted prevalence = 4.0%). The prevalence of variables associated with running out of food at *p* < 0.1 are shown in Table 1. The table presents both the unweighted and weighted prevalences with 95% confidence limits and *p* values for ‘running out of food’. A total of 17,682 correspondents had information for all the variables on Table 1 and this was the sample used to run the multivariable weighted logistic regressions.

Table 2 presents the primary multivariable weighted logistic regression that was used as a basis to create the path analysis. The odds ratios for interaction terms that are presented in the path are estimates based on the results of the regression which either attenuates the effect or enhances the effect. This model showed good fit with the data (χ^2^ = 11.02, *p* = 0.27) and was used as the basis of the path diagram.

**Figure 1:**
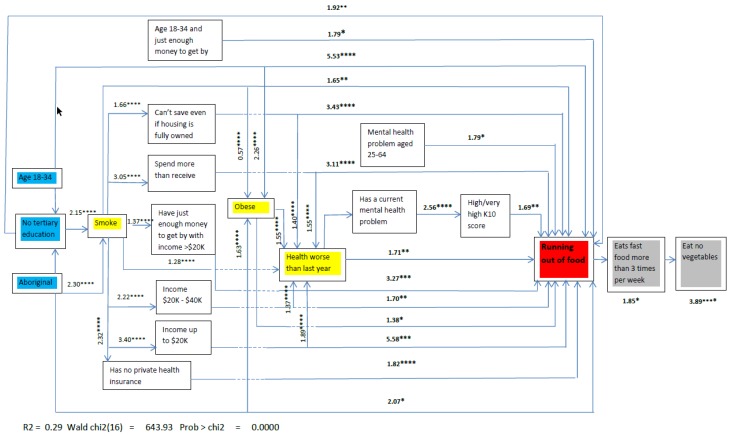
Estimate of probability of eating fast food more than twice a week and eating no vegetables by ‘running out of food’, adjusted using propensity scores: Showing probable outcomes of ‘running out of food’, HWSS 2009–2013. * *p* < 0.05; ** *p* < 0.01; *** *p* < 0.001; **** *p* < 0.0001.

The path to ‘running out of food’ and the associations between variables are shown in Figure 1. The path diagram shows both the main effects and the interaction terms that directly or indirectly influence the primary outcome of ‘running out of food’. The models showed a good fit with the data, both for the variables that are associated with ‘running out of food’ (χ^2^ = 12.75; *p* = 0.17) and the possible consequences of ‘running out of food’ (fast food consumption χ^2^ = 5.48; *p* = 0.71; not eating vegetables χ^2^ = 8.82; *p* = 0.31).

In Figure 1, the red box represents the outcome measure, demonstrating food insecurity, i.e., ‘running out of food’ at least once in the previous twelve months. The blue boxes represent sociodemographics that are not able to be changed or are not easily changed. The yellow boxes represent associations which modify other variables on the path to food insecurity as well as being directly associated with food insecurity. The grey boxes represent the hypothesised consequences of food insecurity as informed by the BIC analysis. 

### 3.1. Direct Associations with Food Insecurity 

Of the variables regarded as fixed, only three were directly associated with food insecurity: Age group (18–34 years compared to 35–64 years, odds ratio (OR) = 5.53, *p* < 0.0001), prior education level (no tertiary education compared with tertiary education, OR = 1.92, *p* < 0.01) and Aboriginality (of Aboriginal or Torres Strait Islander origin compared with not, OR = 2.07, *p* < 0.05). With the exception of two variables (which are dependent on ‘running out of food’) all other variables predicted FI. The two variables that were dependent on ‘running out of food’ were frequent fast food consumption, which subsequently predicted eating no vegetables. 

### 3.2. Effect Modifiers in the Food Insecurity Path 

Three variables acted as powerful effect modifiers in the path, shown in the yellow boxes: Smoking, obesity and the perception of worsening health over time (direct association). The size of the effects is shown on the chart as odds rations. To illustrate: Smoking, which is influenced by Aboriginality (OR = 2.30, *p* < 0.0001) and education (OR = 2.15, *p* < 0.0001), has a main effect on ‘running out of food’ (OR = 1.65, *p* < 0.01) and also acts as an effect modifier for perceived spending power and income (OR = 1.65, *p* < 0.01), income (AUD$20–40K OR = 2.22, *p* < 0.0001; up to AUD$20K OR = 3.40, *p* < 0.0001), private health insurance (OR = 1.65, *p* < 0.01) and worsening health status (OR = 1.65, *p* < 0.01). 

Obesity influenced by smoking, where aboriginality and younger age has a main effect (OR = 1.38, *p* < 0.05) acts as an effect modifier for worsening health (OR = 1.55 *p* < 0.0001). Worsening health is influenced by smoking, obesity, low income, discretional income, and money problems, which are defined here as any income perceived to be less than needed. It has a main effect (OR = 1.71, *p* < 0.01) and acts as an effect modifier on mental health (OR = 1.71, *p* < 0.01). 

Independent associations between younger age and spending power, low income and spending power, money problems, and mental health problems for older respondents are all directly associated with ‘running out of food’. Other direct effects include not having private health insurance, having a low income, discretional income and mental health. Mental health also has an indirect effect mediated by high psychological distress, with the score measured by the K10 scale.

Respondents who are younger or who have very low incomes are more than five times as likely to report ‘running out of food’ compared with older age respondents and those with higher incomes. Respondents with money problems, low discretional income, and those with both low income and low discretional income are more than three times as likely to report ‘running out of food’ compared with respondents who don’t have money problems and higher income as well as higher discretional spending power. 

### 3.3. Adjustment for Covariates and Indicators of Hypothetical Causality 

Using iterative propensity score weighted analyses, three areas of the path were tested for hypothetical causality with regard to food insecurity: Having a low annual household income, an inadequate perceived discretional income and obesity. Additionally, two areas were tested for possible causality due to food insecurity: Eating fast food more than twice a week and not eating any vegetables. Table 3 shows the results for the link between food insecurity and having an annual household income of up to AUD$20,000, being able to save versus other discretional income categories and being obese versus not.

The first line of Table 3 shows the difference in the probability of ‘running out of food’ for the population with low income compared with those with a higher income. The second line of the table shows the probability for a reference higher income group ‘running out of food’. The overall probability of ‘running out of food’ for the low income group is the sum of the two coefficients (e.g., 0.038 + 0.028 = 0.066). The next lines of Table 3 show the difference between the population and the reference category(ies) with which they are being compared for the independent variables: Perceived discretional income and obesity. The Supplementary Table S1 shows the full model for incomes above and below $20,000.

Table 4 shows the difference between the population and the reference category(ies) with which they are being compared for eating fast food three or more times a week and not eating vegetables. Eating fast food three or more times a week precedes eating no vegetables (as assessed using BIC in the path analysis). 

## 4. Discussion

This analysis using a population-based survey resulted in the description of a plausible and quantified pathway to food insecurity, as well as measurement of its dietary impacts. The findings support the hypothesis that food insecurity, measured by whether or not a household ran out of food in the last year, appears to more strongly precede the poor diet indicators of eating fast food three or more times a week and eating no vegetables. While this hypothesis has been proposed previously [51], this proof-of-concept study is the first to quantify both the relative importance of the factors within the feedback loop to food insecurity and the complex nature of the factors leading to it. 

The odds of ‘running out of food’ were higher for younger adults, those without tertiary education and Aboriginal people (odds ratios of 5.53, 2.15 and 2.3 respectively). These findings are consistent with the findings of the Australian national dietary survey which found that the prevalence of food insecurity was higher in Aboriginal populations compared to non-Indigenous Australians (22% compared to 3.7%) [52]. A UK study of 10,452 adults found that different socioeconomic indicators predicted dietary intake, for example, economic access to food, educational attainment and age were related to fruit and vegetable intake and diet costs [53]. The study also showed that dietary costs were not equally important in the causal pathway between socioeconomic position, suggesting that health and diet may be a factor when allocating funding for food [53]. 

The risk of running out of money for food has been associated with one-off financial stressors such as medical or other expenses due to unexpected events [54] and the price of food has been shown to contribute to food stress among low income families [55]. This current research highlights many other interrelated and potentially changeable factors. Saving ability, at any income level, appears to protect against food insecurity. Financial over-commitment is particularly relevant during times of economic downturn, as has occurred in Western Australia over recent years. The financial stress of housing costs may contribute to food insecurity as people can no longer afford their mortgage or rent, leading to money for food, a discretionary expense, potentially being sacrificed and instead put towards covering housing costs. 

Attitudes toward income inequality and how poor people manage their money and cope in stressful situations are underpinned by cultural beliefs, related to blame, plight and privilege [56]. Although most Canadians were willing to accept differences in income related health inequalities such as food insecurity, they were less willing to attribute health inequalities to differences in personal health practices and coping skills [56]. The current study found increased odds of smoking, eating fast food and obesity associated with running out of money to buy food, independent of income level. Similar associations with food insecurity, perceived financial difficulty, smoking, lower fruit and vegetable intake and higher discretionary food intake were found in a representative sample of French households [57].

There are significant associations with not having private health insurance and feeling a lack of control over one’s own health, both of which have adjusted odds ratios of 1.9. There is also an increased likelihood of food insecurity associated with social, mental and physical disadvantage as noted in other studies [58]. The number of factors on the path support the notion of deprivation amplification [59]. The results also show that quantification of the degree and direction of effects associated with food insecurity is possible. This quantification lends support to the concept that there may be causality between some of the variables on the path. 

According to the theory underlying propensity score methodology as developed by Rosenbaum and Rubin [60], adjustment for propensity scoring removes the influence of confounding by multiple covariates and provides plausible evidence for causation for cross-sectional studies, even though one cannot ascribe any statistical significance to the findings [61]. The results from this current study suggest evidence of a hypothetical causal relationship within the path for the variables presented. The direction of effect is the relative strength of the association, so for example, while obesity may follow FI, it more strongly precedes it. The same applies to eating no vegetables and fast food consumption, which also precede but more strongly follow FI as shown in the path diagram. This is one possible path to FI which provides opportunities to investigate the mechanisms underlying its effects and the mediation illustrated in the path diagram. Other paths to FI can further investigate latent effects such as dietary eating patterns [10]. Unhealthy eating patterns are associated with similar sociodemographics found in the FI pathway of this study [62].

There is value for policy decision makers in quantifying both the relative importance of a range of associations with food insecurity and constructing a path to food insecurity. The path diagram uses a population dataset with enough statistical power to allow for a subsequent investigation of possible causal links, highlighting the need to address the determinants of food insecurity as well as considering the consequences. The complex nature of the path also adds weight to the need for inter-sectoral collaborations to address the various determinants of food security. The results from the path diagram support the need for a system level policy to address this [63,64]. For example, obesity, which in the path diagram more strongly precedes FI has been shown also to follow it [62,65], suggesting the need for a policy that addresses both obesity as well as FI in tandem rather than as a separate policy for each. 

The strength of this analysis is that the population survey and large sample size enabled the complex analysis to establish a proof-of-concept study to be undertaken. As with all survey data, there are limitations associated with this research, including some level of non-response and the self-reported nature of the data. The use of sample design weights incorporated in iterative proportional fitting (IFP), also known as ‘raking’, allowed for adjustment of over and under representation of age, gender and area of residence within the sample. This weighting was also incorporated into the multivariable regression models. The one question measure of food insecurity, based on ‘running out of food’ and not being able to afford more, does not measure the extent and experience of food insecurity, nor did the self-reported brief diet questions measure actual dietary intake. However, that was not the purpose of this investigation, which was to explore the complex mix of influences leading to FI. A further limitation was the omission of questions relating to attitudinal and lifestyle behaviours which would have allowed for the creation of a model that included these modifiable attributes. 

## 5. Conclusions

The findings support evidence that decisions about food insecurity are complex and interactive, with a variety of factors contributing to the issue. While the single measure of FI cannot be considered adequate to fully estimate the prevalence of FI, the proof-of-concept using this single measure showed expected associations and quantified the effects of ‘running out of food’ over a range of determinants, such as income and physical and mental wellbeing. The path diagram presented suggests that a wider approach to bringing about change in access and use of food needs to be considered. The findings highlight the need to focus policy effort on mitigating the social determinants of food insecurity and the potential complexity of the pathways to food insecurity. This requires a system approach to policy development for FI and we could encourage policy makers and researchers to use this methodology to explore and quantify the complex relationships leading to food insecurity.

## Figures and Tables

**Table 1:** The unweighted and weighted prevalences of ‘running out of food’ by sample characteristics, HWSS 2009–2013 (n = 21,705 ^a^).

Demographic Variables	Unwght %	Wght %	95% CI	*p*
18–24	7.8	8.0	[6.5, 9.9]	
25–34	5.1	4.9	[3.8, 6.2]	
35–44	3.6	3.2	[2.6, 4.0]	
45–54	2.8	2.4	[1.9, 2.9]	
55–64	2.0	1.6	[1.3, 2.0]	<0.0001
Tertiary education	1.4	1.9	[1.3, 2.6]	
Less than tertiary education	3.8	4.7	[4.1, 5.3]	<0.0001
Employed	2.2	2.9	[2.5, 3.4]	
Unemployed	11.4	12.6	[9.0, 17.7]	
Home duties	4.3	5.2	[4.0, 6.7]	
Retired	2.3	2.0	[1.3, 3.0]	
Student	7.5	7.1	[4.8, 10.3]	
Unable to work	17.3	17.6	[13.2, 23.0]	<0.0001
Annual household income: over AUD $40,000	1.7	2.4	[2.0, 2.9]	
Annual household income: AUD $20,001–$40,000	7.0	9.6	[7.6, 12.2]	
Annual household income: up to AUD $20,000	15.0	17.8	[14.2, 22.2]	<0.0001
Spend left over money or save some per pay	1.1	1.7	[1.4, 2.0]	
Just enough money to get by per pay	10.6	12.5	[10.7, 14.5]	
Not enough money to get by per pay	17.5	19.0	[15.1, 23.6]	<0.0001
Not aboriginal	3.1	3.8	[3.4, 4.2]	
Aboriginal	12.5	15.0	[9.8, 22.1]	<0.0001
Adults living with others	2.8	3.7	[3.3, 4.2]	
Adults living alone	6.0	6.4	[5.2, 7.8]	<0.0001
Born outside Australia	2.8	2.9	[2.3, 3.7]	
Born in Australia	3.5	4.4	[3.9, 5.0]	0.002
Rents or pays mortgage	4.1	4.6	[4.3, 5.0]	
No mortgage or Government subsidized housing	2.5	3.1	[2.7, 3.4]	0.0003
SEIFA ^b^ Quintile 5 (least disadvantaged area)	2.4	2.9	[2.3, 3.6]	
SEIFA Quintiles 3,4 (less disadvantaged areas)	3.4	4.5	[3.9, 5.3]	
SEIFA Quintiles 1,2 (most disadvantages areas)	4.0	5.2	[4.2, 6.4]	<0.0001
Has a health care card	10.3	11.3	[9.7, 13.2]	<0.0001
Doesn’t have private health insurance	7.0	8.3	[7.2, 9.6]	<0.0001
Has asthma	5.7	6.3	[4.7, 8.4]	0.0011
Some cardiovascular condition	5.8	7.4	[4.9, 11.0]	0.0022
Has cancer	4.5	7.0	[4.3, 11.3]	0.0167
Current mental health (depression/anxiety/other)	9.1	9.7	[8.3, 11.4]	<0.0001
Health rated as fair/poor	8.8	8.9	[7.2, 11.0]	<0.0001
Always or often feel a lack of control over health	12.8	13.9	[11.0, 17.3]	<0.0001
Health rated worse than 12 months ago	7.3	9.4	[7.6, 11.6]	<0.0001
High/very high Kessler 10 score	14.1	14.8	[12.4, 17.6]	<0.0001
BMI 30 or more (in obese range)	4.3	5.2	[4.4, 6.1]	<0.0012
Currently smoking	7.1	8.5	[7.0, 10.3]	<0.0001
Does no leisure time physical activity	4.4	5.5	[4.0, 7.5]	0.0447
Spends four or more hours sitting in leisure time	6.4	7.6	[5.8, 9.8]	<0.0001
Eats ‘fast food’ ^c^ three or more times a week	9.1	11.9	[8.3, 17.0]	<0.0001
Uses full fat milk	4.6	5.7	[4.9, 6.7]	<0.0001
Doesn’t eat any fruit	6.3	6.4	[4.5, 9.1]	
Eats less than two serves of fruit daily	3.4	4.2	[3.6, 4.9]	
Eats two or more serves of fruit daily	2.7	3.3	[2.8, 4.0]	0.0030
Doesn’t eat any vegetables	15.0	14.9	[6.5, 30.4]	
Eats less than five serves daily	3.3	4.0	[3.6, 4.5]	
Eats five or more serves daily	2.3	2.6	[1.7, 3.9]	<0.0012

^a^ Sample with no missing values for each sociodemographic variable: Age (n = 21,705); education (n = 21,659); employment status (21,556); income (n = 17,964); perceived spending power (n = 20,959); aboriginal or not (n = 21,694); born in Australia or not (n = 21,704); living arrangements (n = 21,687); own or mortgage/rent (n = 21,705) SEIFA (n = 21,705); ^b^ SEIFA is an index of relative social disadvantage by area of residence [43] usually presented as quintiles which have been grouped into three levels of social disadvantage for this study; ^c^ Fast food is operationally defined as take away food such as burgers, pizza, chicken or chips from places like McDonalds, Hungry Jacks, Pizza Hut or Red Rooster.

**Table 2:** Weighted multivariable logistic regression for associations with running out of food including interaction terms, HWSS 2009–2013 (n = 17,638 ^a^) ^b^.

Main Effects	Odd Ratio (95% CI)	*p*
35 over	Ref	
18–34 years	5.29 (3.65, 7.65)	0.000
Has tertiary education	Ref	
Does not have tertiary education	1.87 (1.38, 2.54)	0.000
Not Aboriginal	Ref	
Aboriginal	2.07 (1.34, 3.2)	0.001
Household income over $40,000	Ref	
Household income $20,000 to $40,000	1.65 (1.29, 2.1)	0.000
Household income under $20,000	5.28 (3.91, 7.13)	0.000
Can save a bit of money	Ref	
Just enough money to get by	1.08 (0.69, 1.71)	0.730
Not enough money to get by	3.11 (2.17, 4.46)	0.000
Has private health insurance	Ref	
Has no private health insurance	1.80 (1.46, 2.22)	0.000
Does not have doctor diagnosed mental health problem	Ref	
Has a doctor diagnosed mental health problem	2.56 (1.96, 3.35)	0.000
Low or moderate Kessler 10 score	Ref	
High or very high Kessler 10 score	1.65 (1.31, 2.06)	0.000
Health same or better than same time previous year	Ref	
Health worse or much worse than same time previous year	1.70 (1.37, 2.09)	0.000
Does not smoke	Ref	
Smokes	1.58 (1.29, 1.93)	0.000
Is not in Body Mass Index obese range	Ref	
Is in Body Mass Index obese range	1.44 (1.18, 1.76)	0.000
Eats some vegetables daily	Ref	
Eats no vegetables daily	2.40 (1.34, 4.3)	0.003
Eats fast foods less than three times a week	Ref	
Eats fast foods three or more times a week	1.83 (1.11, 3.01)	0.018
Interaction terms		
Has just enough money to get by ^#^ age 18–24 years	0.56 (0.35, 0.91)	0.019
Has a mental health problem ^#^ age 18–24 years	0.52 (0.31, 0.86)	0.010
Housing whether or not owned or rented ^#^ Not enough or just enough money to get by	3.35 (2.41, 4.65)	0.000
Household income under $20,000 ^#^ Not enough or just enough money to get by	3.05 (1.94, 4.80)	0.000

^a^ Logistic reduced the estimation sample as it ran with post stratification adjustment (accounting for new weighted estimation sample); ^b^ This is the basic model used to determine the direction of effect. Two further models were then produced: One for associations preceding running out of food and one for associations following running out of food. The odd ratios in the path diagram were taken from these two models; ^#^ Denotes interaction terms between variables: Odds ratios less than 1 attenuate the effect and odds ratios greater than 1 enhance the effect.

**Table 3:** Estimate of probability of food insecurity (‘running out of food’ and not being able to afford more) by income, discretional income and obesity, adjusted using propensity scores: Showing probable antecedent factors of ‘running out of food’, HWSS 2009–2013.

Outcome: ‘Running out of Food’ at Least Once in the Previous Twelve Months	Coef.	95%	CI	Robust Std. Err	Z	*p*
**Annual household income**						
Average effect when income <$20,000	0.038	0.013	0.063	0.013	3.02	0.003
Probability if income is >$20,000	0.028	0.025	0.03	0.001	19.33	<0.001
**Discretional income**						
Difference between spend left over vs. able to save	0.023	0.014	0.033	0.005	4.85	<0.001
Difference between just enough vs. able to save	0.056	0.046	0.067	0.005	10.48	<0.001
Difference between not enough vs. able to save	0.066	0.048	0.083	0.009	7.38	<0.001
Average probability of outcome for those able to save	0.012	0.009	0.014	0.001	9.05	<0.001
**Obesity**						
Difference in probability when obese	0.008	0.003	0.013	0.003	3.15	0.002
Average probability of outcome if not obese	0.029	0.026	0.032	0.002	17.88	<0.001

vs = versus.

**Table 4:** Estimate of probability of eating fast food more than twice a week and eating no vegetables by ‘running out of food’, adjusted using propensity scores: Showing probable outcomes of ‘running out of food’, HWSS 2009-2013.

Outcome:	Coef.	95%	CI	Robust Std. Err	Z	*p*
**Eats fast food more than three times a week**						
Difference in probability of ‘running out of food’ vs. not	−0.007	−0.013	−0.0002	0.003	−2.03	0.042
Average probability of outcome when didn’t run out	0.019	<0.001	0.017	0.001	17.83	<0.001
**Eats no vegetables**						
Difference in probability of fast food >2 times weekly	0.029	0.007	0.051	0.011	2.61	0.009
Average probability of FI when fast food <3 times weekly	0.006	0.005	0.007	0.001	10.32	<0.001

## Supplementary Materials

The following are available online at http://www.mdpi.com/1660-4601/15/12/2620/s1, Table S1: Illustrating the full model of propensity scoring for incomes above and below $20,000.
