# Supplementary material for: Using Cross-Sectional Data to Identify and Quantify the Relative Importance of Factors Associated with and Leading to Food Insecurity
Source: Int J Environ Res Public Health. 2018 Nov 22;15(12):2620. doi: 10.3390/ijerph15122620 (PMC6313516; doi:10.3390/ijerph15122620)
Supplement: Supplementary file 1 [file ijerph-15-02620-s001.pdf]

Supplementary Table illustrating the full model of propensity scoring for incomes above and below $20,000

	Coef.	95% CI		Robust Std. Err.	z	*p*
Average effect when income is less than $20,000	0.038	0.013	0.063	0.013	3.020	0.003
Probability of running out of food if income is higher than $20,000	0.028	0.025	0.030	0.001	19.330	<0.001

**Covariates for not running out of food**	**Coef.**	**95% CI**		**Robust Std. Err.**	**z**	***p***
Can save regularly vs can save lots	0.217	-0.687	1.122	0.462	0.470	0.638
Can save a bit sometimes vs can save lots	1.160	0.303	2.017	0.437	2.650	0.008
Some left but spent vs can save lots	2.262	1.397	3.126	0.441	5.130	<0.001
Just enough to get by vs can save lots	2.990	2.159	3.821	0.424	7.050	<0.001
Spending more than getting vs can save lots	3.514	2.659	4.369	0.436	8.060	<0.001
Of aboriginal origin vs not	0.825	0.286	1.363	0.275	3.000	0.003
Male vs female	-0.167	-0.405	0.071	0.122	-1.370	0.170
Age In years	-0.055	-0.066	-0.045	0.005	-10.380	<0.001
Rented from government vs paying a mortgage	-0.047	-0.553	0.459	0.258	-0.180	0.856
Rented privately vs paying a mortgage	0.368	0.081	0.654	0.146	2.510	0.012
Fully owned vs paying a mortgage	-0.067	-0.357	0.223	0.148	-0.450	0.650
Other living arrangement vs paying a mortgage	-0.181	-0.874	0.512	0.354	-0.510	0.609
Doesn't have a tertiary education vs does	0.524	0.193	0.855	0.169	3.100	0.002
Doesn't have private health insurance vs does	0.690	0.457	0.924	0.119	5.790	<0.001
Has a mental health condition vs doesn't	0.469	0.202	0.735	0.136	3.450	0.001
Moderate psychological distress vs low	0.637	0.351	0.923	0.146	4.370	<0.001
High psychological distress vs low	1.115	0.773	1.456	0.174	6.390	<0.001
Very high psychological distress vs low	1.180	0.733	1.626	0.228	5.170	<0.001
Smokes	0.421	0.179	0.664	0.124	3.400	0.001
Is obese	0.307	0.083	0.530	0.114	2.690	0.007
Constant	-4.555	-5.507	-3.603	0.486	-9.380	<0.001

**Covariates for running out of food**	**Coef.**	**95% CI**		**Robust Std. Err.**	**z**	***p***
Can save regularly vs can save lots	-0.209	-2.233	1.816	1.033	-0.2	0.84
Can save a bit sometimes vs can save lots	-0.258	-2.206	1.691	0.994	-0.26	0.795
Some left but spent vs can save lots	0.006	-2.077	2.088	1.063	0.01	0.996
Just enough to get by vs can save lots	1.003	-0.864	2.871	0.953	1.05	0.292
Spending more than getting vs can save lots	1.752	-0.117	3.622	0.954	1.84	0.066
Of aboriginal origin vs not	0.353	-0.431	1.137	0.4	0.88	0.378
Male vs female	-0.308	-0.717	0.102	0.209	-1.47	0.141
Age In years	-0.026	-0.042	-0.009	0.008	-3.07	0.002
Rented from government vs paying a mortgage	0.16	-0.406	0.725	0.289	0.55	0.58
Rented privately vs paying a mortgage	0.383	-0.195	0.962	0.295	1.3	0.194
Fully owned vs paying a mortgage	-0.473	-1.015	0.068	0.209	-1.47	0.141
Other living arrangement vs paying a mortgage	0.107	-0.84	1.055	0.483	0.22	0.824
Doesn't have a tertiary education vs does	0.529	-0.157	0.35	1.51	0.131	-0.157
Doesn't have private health insurance vs does	0.703	0.275	1.131	0.218	3.22	0.001
Has a mental health condition vs doesn't	0.867	0.463	1.271	0.206	4.21	<0.001
Moderate psychological distress vs low	0.782	0.292	1.273	0.25	3.13	0.002
High psychological distress vs low	1.041	0.523	1.558	0.264	3.94	<0.001
Very high psychological distress vs low	1.286	0.72	1.851	0.289	4.46	<0.001
Smokes	0.331	-0.054	0.717	0.197	1.68	0.092
Is obese	0.365	-0.003	0.733	0.188	1.94	0.052
Constant	-3.375	-5.41	-1.34	1.038	-3.25	0.001

Covariates for running out of food after adjustment based on propensity scoring	Coef.	95% CI		Robust Std. Err.	z	*p*
Can save regularly vs can save lots	0.724	0.261	1.188	0.236	3.060	0.002
Can save a bit sometimes vs can save lots	1.687	1.234	2.140	0.231	7.300	<0.001
Some left but spent vs can save lots	1.848	1.352	2.344	0.253	7.300	<0.001
Just enough to get by vs can save lots	2.700	2.246	3.154	0.232	11.660	<0.001
Spending more than getting vs can save lots	3.204	2.723	3.685	0.245	13.070	<0.001
Male vs female	-0.333	-0.471	-0.195	0.070	-4.730	<0.001
Age In years	0.038	0.030	0.046	0.004	9.250	<0.001
Rented from government vs paying a mortgage	2.054	1.810	2.297	0.124	16.540	<0.001
Rented privately vs paying a mortgage	1.113	0.892	1.334	0.113	9.880	<0.001
Fully owned vs paying a mortgage	1.208	1.026	1.389	0.093	13.040	<0.001
Other living arrangement vs paying a mortgage	1.327	0.902	1.752	0.217	6.120	<0.001
Doesn't have a tertiary education vs does	0.521	0.327	0.715	0.099	5.270	<0.001
Doesn't have private health insurance vs does	1.128	0.990	1.266	0.070	16.000	<0.001
Has a mental health condition vs doesn't	0.188	0.018	0.359	0.087	2.160	0.031
Moderate psychological distress vs low	0.294	0.125	0.464	0.086	3.400	0.001
High psychological distress vs low	0.707	0.484	0.930	0.114	6.220	<0.001
Very high psychological distress vs low	0.926	0.640	1.211	0.146	6.360	<0.001
Constant	-8.160	-8.788	-7.532	0.320	-25.470	<0.001

The first line of the first table shows the difference in the probability of running out of food for the population with low income compared with those with a higher income. The second line of the table shows the probability for reference higher income group running out of food. The overall probability of running out of food for the low income group is the sum of the two coefficients (e.g. 0.038+0.028=0.066). The other tables show the model for the covariates of not running out of food and running out of food and the final part of the table is the ‘propensity model’ which shows which variables are associated with having an annual household income up to $20,000 (chosen to illustrate the model).
